# Efficient and improved synthesis of Telmisartan

**DOI:** 10.3762/bjoc.6.25

**Published:** 2010-03-11

**Authors:** A Sanjeev Kumar, Samir Ghosh, G N Mehta

**Affiliations:** 1 Applied Chemistry Department, Sardar Vallabhbhai National Institute of Technology, Surat-395 007, India

**Keywords:** antihypertensive drug, oxazoline hydrolysis, Suzuki coupling, Telmisartan

## Abstract

An efficient synthesis of the angiotensin II receptor antagonist Telmisartan (**1**) is presented involving a cross coupling of 4-formylphenylboronic acid **10** with 2-(2-bromophenyl)-4,4-dimethyl-2-oxazoline (**11**) as the key step (90% yield). The benzimidazole moiety **15** was constructed regioselectively via a reductive amination-condensation sequence, replacing the alkylation of the preformed benzimidazole step in the previously published route. This methodology overcomes many of drawbacks associated with previously reported syntheses.

## Introduction

Telmisartan (**1**) is an angiotensin II receptor antagonist useful in the treatment of hypertension, heart diseases, heart attack, and bladder diseases [1–3]. Telmisartan is currently available in the market as an antihypertensive drug [4] under the brand name of Micardis^®^.

**Figure 1:**
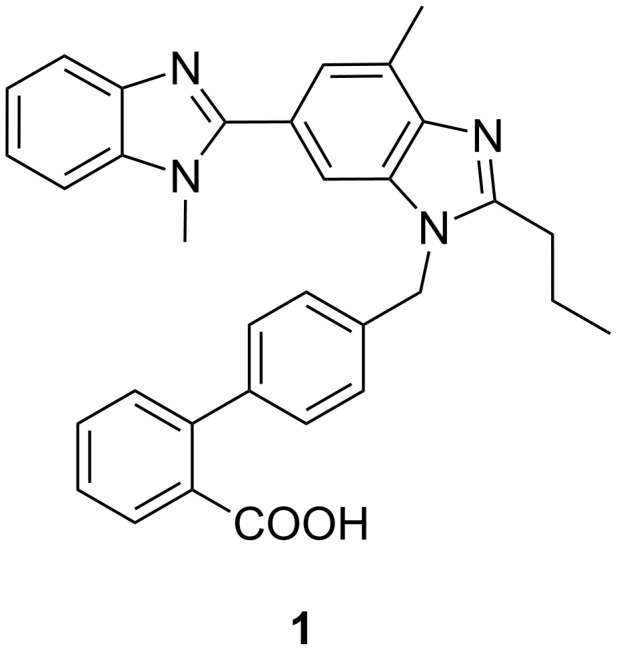
The angiotensin II receptor antagonist Telmisartan.

Essential hypertension is a major risk factor in cardiovascular diseases and is responsible for one-third of global deaths. Most antihypertensive drugs interact with the renin-angiotensin system (RAS), which is the central regulator of blood pressure and electrolyte homeostasis. Renin transforms angiotensinogen into the decapeptide angiotensin I, which is converted by the angiotensin conversion enzyme (ACE) into the octapeptide angiotensin II. The latter binds to its angiotensin receptor (AT_1_) and, thereby, becomes a powerful vasoconstrictor. In the early 1990s, Merck introduced the non-peptidic orally active angiotensin II receptor antagonist losartan (Lozaar) as the first member of a new class of antihypertensive drugs called sartans, all of which contain a characteristic *ortho* functionalized biaryl moiety. Telmisartan (**1**, Boehringer Ingelheim, Micardis^®^) (Figure 1) is an important member of this class of top-selling drugs because it has the strongest binding affinity to the AT_1_ receptor, an excellent bioavailability, and a once-per-day dosage.

**Scheme 1:**
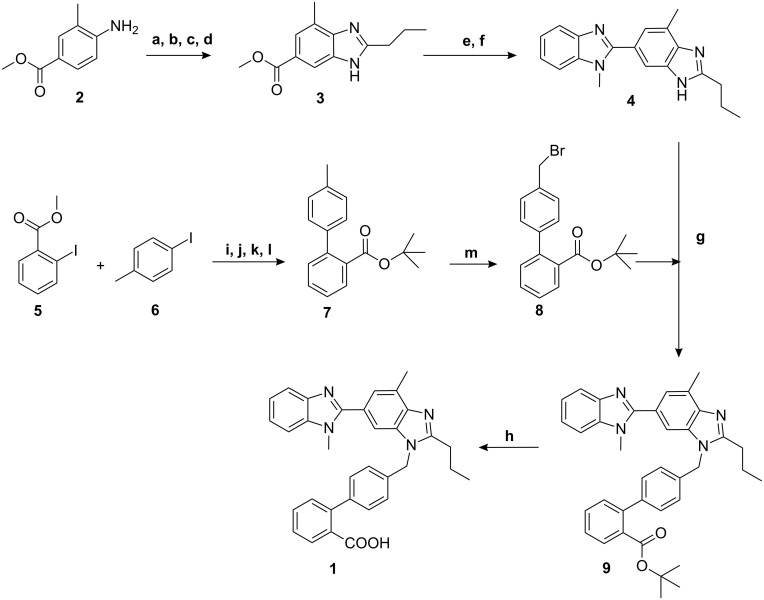
First literature synthesis of Telmisartan (a) *^n^*PrCOCl, C_6_H_5_Cl, 100 °C (b) HNO_3_/H_2_SO_4_, 0 °C (c) Pd/C, 5 bar, H_2,_ MeOH (d) AcOH, 120 °C, yield: 78% (e) NaOH, MeOH/H_2_O, 100 °C (f) 2-MeNH-C_6_H_4_-NH_2_, PPA, 150 °C, yield: 64% (g) *^t^*BuOK, DMSO, RT (h) TFA, DCM, RT, yield: 42% (i) Cu (5 equiv), 210 °C, (j) HCl, H_2_O, 100 °C (k) (COCl)_2_, DCM, 0 °C, (l) *^t^*BuOK, THF, RT, yield: 9% (m) NBS, (PhCOO)_2_, CCl_4_, 76 °C.

The first total synthesis of Telmisartan as introduced by Ries et al. (Scheme 1) starts with the acylation of 4-amino-3-methylbenzoic acid methyl ester (**2**) with butyryl chloride, followed by nitration, reduction of the nitro group, and subsequent cyclization of the resulting amine to the benzimidazole derivative **3**. After saponification, the free carboxyl group is condensed with *N*-methyl-1,2-phenylenediamine to afford the bis-benzimidazole **4**, which is then alkylated with the 4′-(bromomethyl)-2-biphenylcarboxylic acid *tert*-butyl ester (**8**) to give, after hydrolysis of the ester group, Telmisartan (**1**) in 21% overall yield and with eight steps as the longest sequence [5].

Several improvements to the above reaction sequence have been reported, e.g., the use of KOH instead of potassium *tert*-butoxide in the penultimate step and the use of methanolic HCl solution instead of trifluoroacetic acid in the final step [6]. However, the main shortcomings of the synthesis remained, namely, the unsatisfactory regioselectivity in the alkylation of **8** with **4** and the intricate synthesis of the biaryl intermediate **7**. In the original protocol, the latter was synthesized via an Ullmann coupling of the aryl iodides **5** and **6** using 5 equiv of copper [7]. Modern syntheses of **7** involve cross-couplings of sensitive aryl magnesium [8], zinc [9], or boron [10–11] compounds with alkyl 2-halobenzoates. Since the commercialization of Telmisartan, **7** has become readily available at low cost, so that most subsequent published procedures start from this compound.

In designing an alternative synthesis of Telmisartan our goal was to minimize the use of expensive and hazardous metals, circumvent the bromination step, and increase the overall efficiency of the synthesis. This was accomplished by reversing the order of the major bond disconnections. We realized biaryl synthesis and reductive amination are the key steps, and have the potential to overcome both of these weaknesses.

## Results and Discussion

**Scheme 2:**
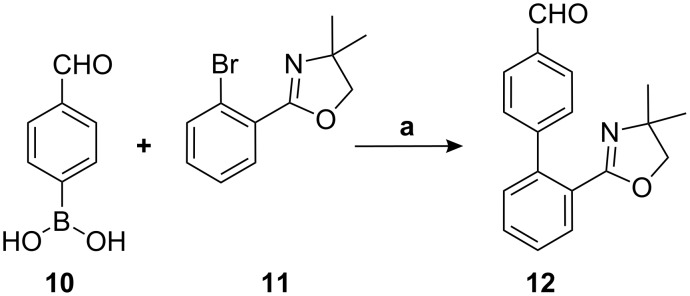
(a) Pd(PPh_3_)_4_, aq Na_2_CO_3_, THF, 12.0 h, 90%.

We identified 4-formylphenylboronic acid (**10**) and 2-(2-bromophenyl)-4,4-dimethyl-2-oxazoline (**11**) [12] as the ideal starting materials for the preparation of the key biaryl intermediate. Thus, Suzuki coupling of 2-(2-bromophenyl)-4,4-dimethyl-2-oxazoline with 4-formylphenylboronic acid in presence of aqueous sodium carbonate and tetrakis(triphenylphosphine)palladium(0) in THF solvent gave 2’-(4,4-dimethyl-4,5-dihydro-1,3-oxazol-2-yl)biphenyl-4-carbaldehyde (**12**) in over 90% yield (Scheme 2).

**Scheme 3:**
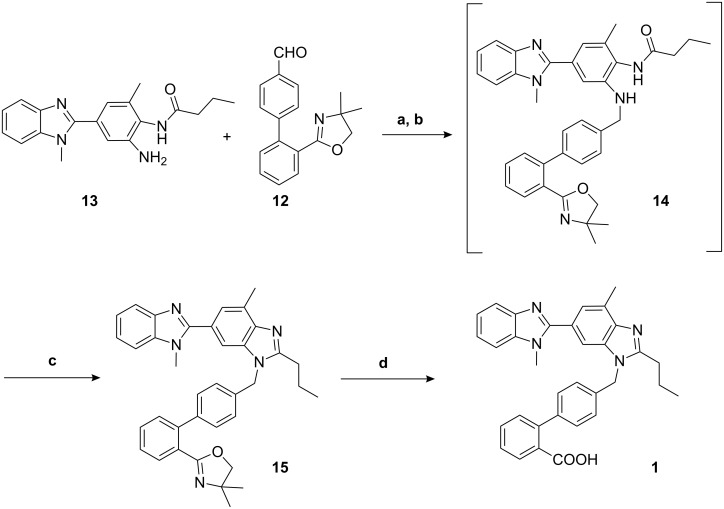
(a) p-TsOH, toluene, 110 °C, 12 h, (b) Pd/C, 7 bar H_2,_ MeOH, 60 °C, 24 h, 100% (c) AcOH, 120 °C, 2 h, 80% (d) Conc. HCl, 100–110 °C, 30 h, 80%.

The reductive amination of the biaryl aldehyde **12** with amine **13** (prepared by the literature procedure [13]) was carried out in the presence of *p*-toluenesulfonic acid in toluene and followed by hydrogenation in methyl alcohol. The resulting amine **14** was not isolated but cyclized in situ to the *n*-propyl benzimidazole **15** in 80% yield in refluxing glacial acetic acid. Finally, cleavage of the oxazoline moiety in **15** by acid afforded Telmisartan (**1**) (Scheme 3).

## Conclusion

In conclusion, a concise and selective synthesis of the antihypertensive drug Telmisartan has been developed, featuring a Suzuki cross-coupling for the construction of the biaryl moiety and a regiospecific reductive amination-condensation sequence for the synthesis of the central benzimidazole.

## Experimental

All solvents and reagents were purchased from the commercial suppliers and used without further purification. All non-aqueous reactions were performed in dry glassware under an atmosphere of dry nitrogen. Organic solutions were concentrated under reduced pressure. Thin layer chromatography was performed on Merck precoated Silica-gel 60F_254_ plates. ^1^H and ^13^C NMR spectra were recorded in DMSO-*d*_6_ and CDCl_3_ using 400 MHz, on a Varian Gemini 400 MHz FT NMR spectrometer. The chemical shifts were reported in δ ppm relative to TMS (tetramethylsilane). The IR spectra were recorded in the solid state as KBr dispersion using Perkin Elmer FT-IR spectrophotometer. The mass spectra were recorded on Shimadzu LCMS-QP 800 LC-MS and AB-4000 Q-trap LC-MS/MS. Melting points were obtained by using the open capillary method and are uncorrected.

**2’-(4,4-dimethyl-4,5-dihydro-1,3-oxazol-2-yl)biphenyl-4-carbaldehyde (12):** To a mixture of 4-formylphenylboronic acid (**10**) (5.0 g, 0.032 mol) and 2-(2-bromophenyl)-4,4-dimethyl-2-oxazoline (**11**) (10.1 g, 0.039 mol) in tetrahydrofuran (50.0 mL), 2 M aqueous sodium carbonate solution (20.0 mL) was added at room temperature. The resulting biphasic solution was degassed with nitrogen gas for 20 min. Tetrakis(triphenylphosphine)palladium(0) (0.25 g) was added and heated to reflux (64 °C). The reaction mixture was maintained under reflux for 12 h. After completion of the reaction, the reaction mixture was cooled to 26 °C and saturated ammonium chloride solution (50 mL) and ethyl acetate (50 mL) added. The organic layer was separated, washed twice with water (50.0 mL), dried over sodium sulfate and evaporated under vacuum. The residue was chromatographed on silica gel eluting with hexane/ethyl acetate 80:20 to give the title compound **12** as an oil (8.0 g, 90%); ^1^H NMR (400 MHz, DMSO-*d*_6_) (δ ppm): 10.0 (1H, s, -CHO), 7.91 (2H, d, *J* = 8.4 Hz, ArH), 7.73 (1H, d, *J* = 8.4 Hz, ArH), 7.48 (2H, d, *J* = 7.8 Hz, ArH), 7.44–7.34 (2H, m, ArH), 7.30 (1H, m, *J* = 7.4 Hz, ArH), 3.80 (2H, s, -CH_2_), 1.12 (6H, s, 2 × -CH_3_); ^13^C NMR (100 MHz, DMSO-*d*_6_) (δ ppm); 28.0, 68.0, 78.9, 128.0, 128.5, 129.5, 129.6, 130.4, 130.5, 131.2, 135.3, 140.2, 147.0, 161.8, 193.2.; MS (*m/z*): 280 [M^+^ + 1].

**3'-{[2'-(4,4-dimethyl-4,5-dihydro-1,3-oxazol-2-yl)biphenyl-4-yl]methyl}-1,7'-dimethyl-2'-propyl-1*H*,3'*H*-2,5'-bibenzimidazole (15)**: A mixture of **13** (4.0 g, 0.01 mol), **12** (3.5 g, 0.01 mol), and *p*-TsOH (0.21 g, 0.001 mol) was suspended in toluene (40 mL) under nitrogen, and the mixture refluxed for 16 h and then concentrated. The residue was diluted with methanol (40 mL) and transferred to a stainless steel autoclave. Palladium on charcoal (10%, 1.0 g) was added and the reaction mixture stirred under H_2_ pressure (7 bar) for 24 h at 60 °C. After cooling to room temperature and filtration, the filter cake was rinsed with ethyl acetate (3 × 50 mL). The filtrate was washed with water, and the aqueous layer basified to pH 10 with aqueous ammonia and extracted with ethyl acetate (2 × 50 mL). The combined organic layers were dried over MgSO_4_ and concentrated. The crude amine **14** was diluted with glacial acetic acid (40 mL), the resulting solution refluxed for 2 h and then concentrated. Water (150 mL) was added to residue. The product was extracted twice with ethyl acetate (2 × 50 mL) and evaporated under vacuum at 55 °C. The residue was triturated with *n*-hexane (40 mL) to yield a solid which was removed by filtration and dried at 50–55 °C for 3–4 h to afford **15** as a white crystalline powder (yield 5.7 g, 80% yield); melting point 191–193 °C; IR (KBr, cm^-1^) 1630 (C=N); HRMS *m/z* calculated for C_37_H_37_N_5_O – 568.7225 [M + 1], found – 568.7222; ^1^H NMR (400 MHz, CDCl_3_) (δ ppm): 7.78 (1H, d, *J* = 8.0 Hz, ArH), 7.68 (1H, m, *J* = 8.0 Hz, ArH), 7.47–7.26 (10H, m, ArH), 7.07 (2H, m, *J* = 8.0 Hz, ArH), 5.45 (2H, s, -CH_2_), 3.82 (3H, s, -CH_3_), 3.58 (2H, s, -CH_2_), 2.97 (2H, t, *J* = 7.6 Hz, -CH_2_), 2.74 (3H, s, -CH_3_), 1.92 (2H, m , *J* = 7.6 Hz, -CH_2_), 1.29 (6H, s, 2 x -CH_3_), 1.04 (3H, t, *J* = 7.6 Hz, -CH_3_); ^13^C NMR (100 MHz, CDCl_3_) (δ ppm): 13.9, 16.7, 21.6, 27.6, 29.6, 31.6, 46.9, 67.2, 79.0, 108.8, 109.2, 119.3, 122.1, 122.2, 123.5, 123.6, 125.6, 127.0, 127.2, 128.8, 129.1, 129.7, 129.9, 130.2, 134.4, 134.8, 136.4, 140.6, 140.8, 142.6, 142.8, 154.2, 156.2, 163.1.

**4’-[(1,7’-dimethyl-2’-propyl-1*H*,3’*H*-2,5’-bibenzimidazol-3’-yl)methyl]biphenyl -2-carboxylic acid (1)**: A mixture of **15** (4.0 g, 0.007 mol) and concentrated hydrochloric acid (40 mL) was heated at reflux (100–110 °C) for about 30 h. The reaction mass was cooled to 0–5 °C. Sodium hydroxide solution (20%) was added until the pH of the reaction mixture was 9–10 and then stirred at room temperature for a further 2 h. The resulting solid was removed by filtration and washed with water (50 mL). The wet cake was dissolved in a mixture of water (60 mL) and acetonitrile (20 mL) and then heated to 60–65 °C. The pH of the resulting clear solution was adjusted to 5.0–5.5 with 5% acetic acid, and stirring continued for 2 h. The precipitated solid was filtered and washed with water (50 mL). After drying at 70–75 °C for 4–5 h under a vacuum Telmisartan (**1**) was obtained as a white crystalline powder (yield 2.9 g, 80%); melting point: 260–262 °C (lit [6] mp 260–262 °C); IR (KBr, cm^-1^) 2300–3500 (broad), 1680 (C=O); HRMS *m/z* calculated for C_33_H_30_N_4_O_2_ – 515.6169 [M + 1], found – 515.6192; ^1^H NMR (400 MHz, CDCl_3_) (δ ppm): 12.8 (1H, s, -COOH), 8.42 (1H, d, *J* = 8.0 Hz, ArH), 8.02 (1H, d, *J* = 8.0 Hz, ArH), 7.50–7.26 (8H, m, ArH), 7.20 (2H, d, *J* = 8.0 Hz, ArH), 7.05 (1H, s, ArH), 6.96 (1H, s, ArH), 5.42 (2H, s, -CH_2_), 3.82 (3H, s, -CH_3_), 2.97 (2H, t, *J* = 7.6 Hz, -CH_2_), 2.74 (3H, s, -CH_3_), 1.92 (2H, m, *J* = 7.6 Hz, -CH_2_), 1.04 (3H, t, *J* = 7.6 Hz, -CH_3_); ^13^C NMR (100 MHz, DMSO-*d*_6_) (δ ppm): 13.5, 16.7, 20.6, 27.6, 32.7, 47.1, 51.7, 112.0, 112.7, 114.7, 118.6, 125.3, 125.7, 125.8, 127.0, 127.4, 128.6, 129.3, 130.4, 130.6, 131.5, 132.3, 133.1, 133.2, 133.7, 134.5, 140.2, 140.5, 150.2, 157.3, 168.1.
